# Clinical outcomes in neovascular age-related macular degeneration: a cohort study of patients with care delay due to the COVID-19 pandemic

**DOI:** 10.1038/s41598-023-41497-4

**Published:** 2023-09-08

**Authors:** Timothy M. Janetos, Roya Zandi, David Younessi, Gina Johnson, Amber Randolph, Manjot Gill

**Affiliations:** 1 Department of Ophthalmology, Northwestern University Feinberg School of Medicine, Chicago, IL USA; 2 Department of Ophthalmology, Northwestern University Feinberg School of Medicine, 645 N. Michigan Ave. Suite 440, Chicago, IL 60611 USA

**Keywords:** Macular degeneration, Epidemiology

## Abstract

The COVID-19 pandemic has led to both intentional and unintentional care delay among age-related neovascular macular degeneration (nvAMD) patients. Prior studies have demonstrated that patients who discontinue nvAMD treatment for prolonged intervals are at high risk for vision loss, but less is known regarding shorter-term delay, such as during the height of the pandemic. Previous studies have looked at COVID-19 related delay in care and have shown a loss of visual acuity (VA) among these patients, but studies are limited by short follow-up or insufficient comparisons. This was an observational cohort study of nvAMD patients from March 1, 2019, through July 1, 2021, who experienced care delay. VA was modeled using a linear longitudinal mixed-effects model comparing historic data pre-lockdown to data post-lockdown. Covariates included baseline anatomic variables, demographic variables, and time intervals (treatment interval, delay interval). Secondary anatomic and treatment outcomes were modeled using a multilevel binary logistic regression model. 163 eyes among 116 patients were included. Initial longitudinal mixed-effects models found that although overall VA decreased at a yearly rate, when comparing pre-lockdown and post-lockdown time periods, VA slopes were not statistically different. Single-covariate longitudinal models showed that age, sex, and delay interval significantly affected VA slope. The multivariate longitudinal model found that a longer delay interval significantly decreased rate of VA loss. Multilevel binary logistic regression models showed a significant increase in odds of anti-VEGF treatment, presence of subretinal fluid, and macular hemorrhages in the post-lockdown period. Overall, when compared to historic data, rate of VA loss among our cohort did not vary significantly in pre-versus post-lockdown time periods, although treatment and anatomic variables did worsen post-lockdown suggesting that patients may be appropriately delayed but this comes at the risk of increased need for treatment.

## Introduction

On March 11, 2020, the coronavirus outbreak (COVID-19) was officially declared a pandemic by the World Health Organization and placed an unprecedented burden on the global healthcare system. On March 18, 2020 the American Academy of Ophthalmology (AAO) released guidance on caring for patients during this emerging crisis and recommended ceasing to provide any patient care that was not deemed as either urgent or emergent^1^. On March 27, 2020 the AAO released an additional list of surgical procedures and indications that were considered urgent or emergent^2^. During this time, many outpatient services across all fields of medicine were essentially closed, and ophthalmology was no exception. The full impact of purposely delaying routine care as well as patient hesitancy to seek care for emergent or urgent conditions is still unfolding. Within ophthalmology, multiple reports of decreased care utilization have emerged with both routine and emergency care^3–6^.

Neovascular age-related macular degeneration (nvAMD) is a condition that requires frequent intravitreal injections to preserve vision with a significant treatment burden to maintain sight^7,8^. Treatment of this condition was considered an emergent procedure by the AAO, however, many patients requiring intravitreal injections for nvAMD delayed care due to a variety of factors. Previous literature has noted a high rate of choroidal neovascular membrane reactivation and worse visual acuity outcomes in patients who had long-term discontinuation of anti-VEGF treatment^9–12^. One study showed a significant loss in visual acuity and a 91% reactivation rate among patients who discontinued treatment for at least 3 months^9^. Additionally, a retrospective study of 35 eyes with discontinuation of treatment for 24 months or longer showed a marked deterioration in visual acuity among this cohort compared to matched controls^12^. Nguyen et al. studied patients with previously inactive disease, defined as at least 3 months of inactivity with no further treatment, who had at least 12-month follow-up and noted a reactivation rate of 41% at 1 year and 79% at 5 years^11^. However, the effect of shorter-term care delays such as during the mandated lockdown period are still being elucidated.

Indeed, several studies have attempted to examined COVID-19 era patients who had care delayed, but conclusions are difficult to make as most lack adequate comparison groups or have short follow-ups with only paired-time point comparisons. Additionally, no study has determined which baseline patient characteristics, if any, could influence visual outcomes in patients who had their care delay due to the COVID-19 pandemic. Like many institutions, our ophthalmology department instituted a mandated lockdown period from March 16, 2020, to May 4, 2020, during which many patients additionally self-cancelled care due to hesitancy caused by the emerging pandemic. We aim to model visual acuity among our nvAMD patients using a rich longitudinal dataset with long-term outcomes in order to capture visual acuity outcomes and determine the effect, if any, delay in care had on patients. Furthermore, we aim to determine any predictive baseline factors that may have influenced visual acuity outcome in this cohort. To date, this is the first modeled longitudinal analysis of visual acuity and anatomic outcomes among patients with nvAMD who delayed care due to the COVID-19 pandemic.

## Methods

Northwestern University Institutional Review Board (IRB) approved the study (IRB# STU00212868) with a waiver of consent due to the lack of intervention and minimal risk to patients. This study conformed to the tenets of the Declaration of Helsinki. Study participants were selected from patients who carried an International Classification of Diseases (ICD-10) diagnosis of nvAMD and had at least one cancelled, no-show, or rescheduled retina-related appointment during the mandated lockdown period (March 16, 2020, through May 4, 2020) within our ophthalmology department. Patients with another maculopathy were excluded (e.g., diabetic macular edema (DME), macular hole, etc.). Data were collected on this cohort from March 1, 2019, through July 1, 2021. This enabled 1-years’ worth of historic data to be compared with greater than 1-years’ worth of follow-up data. Encounter-level data were collected for every ophthalmology encounter during the study period and time varying covariates that were collected included best corrected visual acuity (best acuity noted in the chart at that visit whether refracted, pinhole, or corrected) converted to logMAR visual acuity, presence of subretinal fluid, presence intraretinal fluid, presence of geographic atrophy, and presence of macular hemorrhage on fundoscopic examination. Counts fingers visual acuity and hand motion visual acuity were converted to logMAR visual acuity of 2.0 and 3.0, respectively^13^. Of note, an exploratory analysis excluding hand motion or counts fingers visual acuities did not significantly change output within the models developed and therefore were included in the final model. Light perception and no-light perception eyes were excluded.

Baseline characteristics that were not time varying included age, sex, race, anti-VEGF agent (if actively treated), treatment with prior photodynamic therapy (PDT), and smoking status. Patients were further categorized as active treatment (defined as either newly exudative or having received an injection within 16 weeks of the previous encounter prior to the lockdown period), or sporadic treatment/observation. The patient’s treatment interval, if definable, was collected from the patient’s previous encounter prior to the lockdown period by taking the difference between the two treatment encounters prior to lockdown. For example, a patient who attended a treatment encounter prior to lockdown and had an additional anti-VEGF treatment within 16 weeks prior to this encounter would be classified as active treatment with the treatment interval defined by the time difference between these two encounters. Patients with no treatment prior to lockdown or an interval > 16 weeks were classified as sporadic treatment/observation. Delay interval was defined as the time between the patient’s cancelled appointment and when follow-up occurred. Finally, baseline anatomic variables including subretinal fluid status, intraretinal fluid status, and presence of geographic atrophy were defined as the presence of these anatomic characteristics on optical coherence tomography (OCT) or fundoscopic examination at the encounter immediately prior to the clinic closure.

Visual acuity was modeled using a linear longitudinal mixed-effects model. Time was linearly modeled; a quadratic component was tested and not found to be significant. Eyes were nested within patients. A random slope was included at the eye level and slope was fixed at the patient level as the slope was not found to vary significantly at the patient level. Models were tested with a random slope at both levels without significant variation in output. Time varying encounter data was categorized as pre-lockdown (defined as data from March 1, 2019, to March 16, 2020) or post-lockdown (defined as data from March 17, 2020, to July 1, 2021). Interaction terms between pre-lockdown and post-lockdown time periods and the visual acuity slope were used to determine whether the pre- and post-lockdown slopes varied significantly, and therefore represented a significant change in visual acuity after care delay.

Additional covariates and interaction terms between these covariates and visual acuity included baseline anatomic variables, demographic variables, and the time intervals (treatment interval, delay interval). Each covariate was modeled separately to determine the impact of its interaction with time on visual acuity. Covariates with significant interaction effects were included in the final multivariate model. The final multivariate model included age, sex, delay interval, and intraretinal fluid status. All models included a time period variable to account for any possible pre- and post-lockdown differences. After initial analysis, a subsequent sub analysis was performed using t-tests and Spearman’s rho correlation to evaluate the association between delay interval, treatment interval, and treatment status.

Lastly, secondary outcomes of time varying anti-VEGF injections, subretinal fluid status, intraretinal fluid status, and macular hemorrhage status, were modeled using a multilevel binary logistic regression with pre- and post-lockdown time period fixed effects. All analysis was conducted using SPSS version 27.0 (IBM, Armonk, New York, US). Full deidentified dataset and SPSS code is uploaded in the Supplementary Materials.

### Meeting presentation

Presented in part at ARVO 2022 and ASRS 2022.

## Results

A total of 163 eyes from 116 patients were identified for the study. Two eyes did have ophthalmology follow-up during the mandated lockdown period but were included in the study as one had non-retina related follow-up and the second was still delayed with follow-up rescheduled that fell within the mandated lockdown period. 31 eyes (19%) were permanently lost to follow-up and were not seen from May 4, 2020, to the end of the study. Patients of 12 eyes (7%) died during the follow-up period or during the lockdown period. 14 eyes (8.6%) converted to nvAMD during the study period. Encounter data for all identified patients was included in the study and used for model analysis. Baseline characteristics and demographics are shown in Table 1. The majority of patients were female and white. Average delay interval was 79.5 days. Average treatment interval prior to lockdown in those being actively treated was 60.5 days. 53% of patients were classified as active treatment with the rest being observed or treated sporadically.

**Table 1:** Cohort demographics.

Characteristic	N = 163	(%)
Age (years)		
Median	83.8	
Range	57–99	
Gender		
Female	103	63.20%
Male	60	36.80%
Race		
White	144	88.30%
African American	4	2.50%
Asian	1	0.60%
Declined	14	8.60%
Treatment		
Active treatment (injection interval ≤ 16 weeks)	87	53.40%
Observation/sporadic treatment (injection interval > 16 weeks or no definable treatment interval)	76	46.60%
Treatment interval (days)		
Mean	60.5	
Min	17	
Max	234	
Delay interval (days)		
Mean	79.5	
Min	6	
Max	348	
Anti-VEGF agent (if actively treated)		
Bevacizumab	8	4.90%
Aflibercept	80	49.10%
Ranibizumab	18	11.00%
Study drug	2	1.20%
Subretinal fluid at baseline	46	28.20%
Intraretinal fluid at baseline	43	26.40%
Geographic atrophy at baseline	44	27.00%
Prior PDT	8	4.90%
Smoking status		
Never smoker	79	48.50%
Former smoker	80	49.10%
Current smoker	4	2.50%

**Figure 1:**
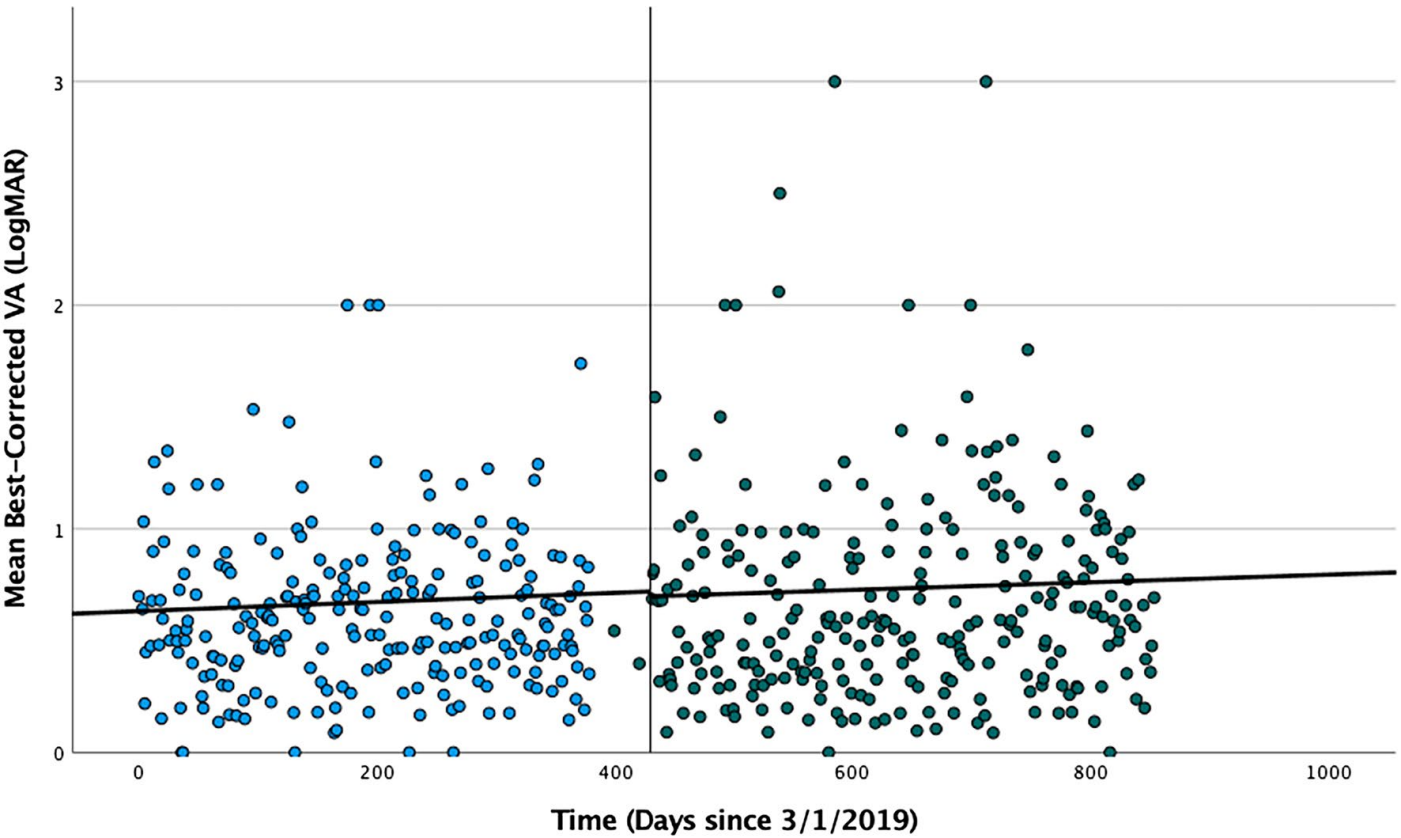
Graphical representation of encounter level mean logMAR visual acuity data segregated by pre- (blue) and post-lockdown (green) time periods (separated by vertical line). Results of the model slope lines are fitted to the pre- and post-lockdown data.

Random slope and fixed effect coefficients in the single-covariate models are shown in Table 2. LogMAR visual acuity slope was positive during the entire study period (0.07154 logMAR/year, 95% CI 0.02336–0.11972, *p* = 0.004), however the interaction term between the pre- and post-lockdown time period was not significant (*p* = 0.703) indicating that visual acuity did not decrease at a different rate in the post-lockdown time period after care delay. Figure 1 shows a graphical representation of the study period encounter data with pre- and post-lockdown model slope lines. Both male sex (0.098185 logMAR/year greater than total slope, 95% CI 0.00584–0.190165, *p* = 0.038) and the delay interval (− 0.03285 logMAR/year per month of delay interval less than total slope, 95% CI − 0.0657 to − 0.002343, *p* = 0.036) significantly affected the visual acuity slope in the single-covariate models indicating that male patients and those that had shorter delay intervals lost visual acuity at a faster rate. Younger patients had significantly better mean visual acuity (0.011642 logMAR/year of age greater than cohort mean visual acuity, 95% CI 0.001516–0.021768, *p* = 0.025), as did patients without intraretinal fluid at the visit prior to the mandated lockdown period (− 0.324881 logMAR less than cohort mean visual acuity, 95% CI − 0.553522 to − 0.096241, *p* = 0.006), but these factors did not influence rate of visual acuity loss over time. Table 3 shows the results of the final multivariate model. In this model, male sex’s effect on visual acuity slope was no longer a significant factor when accounting for delay interval, intraretinal fluid, and age. Delay interval effect on visual acuity slope remained significant in the final model (− 0.03285 logMAR/year per month of care delay, 95% CI − 0.0657 to − 0.002343, *p* = 0.044). Neither the treatment status (active treatment versus sporadic treatment/observation) or treatment interval significantly impacted overall visual acuity or rate of vision loss in this cohort, nor did any anatomic factor predict visual acuity slope (geographic atrophy, subretinal fluid, etc.).

**Table 2:** Single-covariate models showing estimated visual acuity slopes (overall, pre-lockdown, and post-lockdown) as well as effects of patient characteristics on visual acuity mean and slope.

Slope parameter (logMAR/year)	Estimate	Significance	95% Confidence Interval
Visual acuity slope	0.07154	*p* = 0.004	0.02336 to 0.11972
Visual acuity slope pre-lockdown	0.07483	*p* = 0.703^a^	− 0.05074 to 0.07519
Visual acuity slope post-lockdown	0.062415	*p*^a^	–

(a) Patient characteristic	Estimate of effect on visual acuity slope	Significance	95% confidence interval
Age (years)	− 0.004015	*p* = 0.103	− 0.00876 to 0.00073
Sex			
Male	0.098185	*p* = 0.038	0.00584 to 0.190165
Female	0^b^	*p*^b^	–
Race			
White	0.07884	*p* = 0.723	− 0.36281 to 0.52049
African American	− 0.01168	*p* = 0.953	− 0.403325 to 0.379965
Asian	− 0.277035	*p* = 0.305	− 0.81249 to 0.25842
Declined	0^b^	*p*^b^	
Treatment			
Observation/sporadic treatment	− 0.082125	*p* = 0.551	− 0.355145 to 0.190895
Active	0^b^	*p*^b^	
Treatment interval (months)	− 0.01095	*p* = 0.455	− 0.05475 to 0.0219
Delay interval (months)	− 0.03285	*p* = 0.036	− 0.0657 to − 0.002343
Anti-VEGF agent			
Bevacizumab	− 0.047085	*p* = 0.809	− 0.42851 to 0.33434
Aflibercept	− 0.15841	*p* = 0.383	− 0.515745 to 0.19856
Ranibizumab	− 0.185785	*p* = 0.326	− 0.557355 to 0.18542
Study drug	0^b^	*p*^b^	
Subretinal fluid absent at visit prior to lockdown	− 0.06424	*p* = 0.179	− 0.158775 to 0.02993
Intraretinal fluid absent at visit prior to lockdown	0.080665	*p* = 0.126	− 0.02336 to 0.184325
Geographic atrophy absent at visit prior to lockdown	0.038325	*p* = 0.479	− 0.06862 to 0.14527
No prior PDT	0.15841	*p* = 0.129	− 0.047085 to 0.363905
Smoking status			
Never smoker	0.052195	*p* = 0.659	− 0.182135 to 0.28616
Former smoker	0.041245	*p* = 0.728	− 0.193815 to 0.27667
Current smoker	0^b^	*p*^b^	

(b) Patient characteristic	Estimate of effect on mean visual acuity (intercept)	Significance	95% confidence interval
Age (years)	0.011642	*p* = 0.025	0.001516 to 0.021768
Sex			
Male	− 0.131516	*p* = 0.209	− 0.337832 to 0.074799
Female	0^b^	*p*^b^	
Race			
White	− 0.042661	*p* = 0.931	− 1.020453 to 0.935131
African American	0.037126	*p* = 0.935	− 0.872404 to 0.946655
Asian	0.572128	*p* = 0.360	− 0.667780 to 1.812036
Declined	0^b^	*p*^b^	
Treatment			
Observation	0.025863	*p* = 0.929	− 0.550036 to 0.601761
Active	0^b^	*p*^b^	
Treatment interval (days)	0.001324	*p* = 0.357	− 0.001516 to 0.004164
Delay interval (days)	0.000508	*p* = 0.470	− 0.001624 to 0.002639
Anti-VEGF agent			
Bevacizumab	0.002108	*p* = 0.994	− 0.516876 to 0.521092
Aflibercept	0.156395	*p* = 0.487	− 0.285736 to 0.598527
Ranibizumab	0.238790	*p* = 0.336	− 0.248290 to 0.725870
Study drug	0^b^	*p*^b^	
Subretinal fluid absent at visit prior to lockdown	0.063728	*p* = 0.614	− 0.142498 to − 0.269955
Intraretinal fluid absent at visit prior to lockdown	− 0.324881	*p* = 0.006	− 0.553522 to − 0.096241
Geographic atrophy absent at visit prior to lockdown	− 0.107215	*p* = 0.374	− 0.345699 to 0.131269
No prior PDT	− 0.029585	*p* = 0.901	− 0.502798 to 0.443628
Smoking status			
Never smoker	0.015499	*p* = 0.955	− 0.000499 to 0.000784
Former smoker	0.052998	*p* = 0.728	− 0.000531 to 0.000758
Current smoker	0^b^	*p*^b^	

There was not a significant difference in the pre-lockdown and post-lockdown visual acuity slope. Patient characteristics that significantly affected visual acuity slope included sex and delay interval. Patient characteristics that significantly affected visual acuity mean included age, and intraretinal fluid status at the visit prior to lockdown.

(a) Patient characteristics that are categorical (e.g., smoking status, anti-VEGF agent, etc.) are interpreted as the change in visual acuity slope in comparison to the reference category. A reference category is defined as the last category within a given variable. For example, female is the reference category for the sex variable, therefore a coefficient of 0.098185 indicates that males have a greater visual acuity slope (logMAR/year) than females. Continuous variables (e.g., age) are interpreted as the change per relevant unit to visual acuity slope. For example, a patient with a 1-month delay interval would have a 1 × (0.03285) *lower* visual acuity slope (logMAR/year).

(b) Patient characteristics that are categorical (e.g., smoking status, anti-VEGF agent, etc.) are interpreted as the change in visual acuity mean in comparison to the reference category. A reference category is defined as the last category within a given variable. For example, presence of intraretinal fluid is the reference category for the intraretinal fluid variable. Thus, a coefficient of − 0.324881 indicates that patients *without* intraretinal fluid at the visit prior to lockdown had 0.324881 *lower* logMAR visual acuity compared to those who had intraretinal fluid. Continuous variables (e.g., age) are interpreted as the change per relevant unit to visual acuity mean. For example, an 80-year-old patient would have an 80 × (0.011642) *greater* mean logMAR visual acuity compared to cohort mean.

^a^*p*-value shown is estimating a difference in pre- and post-lockdown visual acuity slopes (not significant *p* > 0.05), therefore it is redundant and is not shown.

^b^*p*-values are redundant as these categories are set as the reference category; therefore, estimates are not shown.

**Table 3:** Multivariate model accounting for significant variables found in the single-covariate models showing estimated effects of patient characteristics on visual acuity mean and slope.

(a) Patient characteristic	Estimate of effect on visual acuity slope	Significance	95% confidence interval
Age (years)	− 0.00146	*p* = 0.582	− 0.00657 to 0.00365
Sex			
Male	0.070445	*p* = 0.152	− 0.02628 to 0.16717
Female	0^a^	*p*^a^	–
Delay interval (months)	− 0.03285	*p* = 0.044	− 0.0657 to − 0.002343
Intraretinal fluid absent at visit prior to lockdown	0.048545	*p* = 0.370	− 0.058765 to 0.155855

(b) Patient characteristic	Estimate of effect on mean visual acuity (intercept)	Significance	95% confidence interval
Age (years)	0.007720	*p* = 0.143	− 0.002666 to 0.018107
Sex			
Male	− 0.075951	*p* = 0.476	− 0.286894 to 0.134991
Female	0^a^	*p*^a^	–
Delay interval (days)	0.000349	*p* = 0.746	− 0.001773 to 0.002471
Intraretinal fluid absent at visit prior to lockdown	− 0.249428	*p* = 0.039	− 0.486404 to − 0.012451

When accounting for intraretinal fluid, age, sex, and delay interval, the delay interval was the single significant factor that affected visual acuity slope with a greater interval leading to a lower rate of vision loss. Patients with intraretinal fluid at the visit prior to lockdown had significantly worse visual acuity mean.

(a) Patient characteristics that are categorical (e.g., smoking status, anti-VEGF agent, etc.) are interpreted as the change in visual acuity slope in comparison to the reference variable. A reference variable defined as the last categorical variable is set for each categorical variable. For instance, male sex has a 0.070445 greater visual acuity slope (logMAR/year) than female sex (not significant *p* > 0.05). Continuous variables (e.g., age) are interpreted as the change per variable unit to visual acuity slope. For instance, a patient with a 1-month delay interval would have a 1 × (0.03285) *lower* visual acuity slope (logMAR/year).

(b) Patient characteristics that are categorical (e.g., smoking status, anti-VEGF agent, etc.) are interpreted as the change in visual acuity mean in comparison to the reference category. A reference category is defined as the last category within a given variable. For example, presence of intraretinal fluid is the reference category for the intraretinal fluid variable. Thus, a coefficient of − 0.249428 indicates that patients *without* intraretinal fluid at the visit prior to lockdown had 0.249428 *lower* logMAR visual acuity compared to those who had intraretinal fluid. Continuous variables (e.g., age) are interpreted as the change per relevant unit to visual acuity mean. For example, an 80-year-old patient would have an 80 × (0.007720) *greater* mean logMAR visual acuity (not significant *p* > 0.05).

^a^*p*-values are redundant as these categories are set as the reference category; therefore, estimates are not shown.

Given the counterintuitive effect of delay interval on visual acuity slope, a subsequent analysis of this covariate was performed using t-tests and Spearman’s rho correlation to evaluate the association between delay interval, treatment interval, and treatment status. The sporadic treatment/observation group had a significantly greater mean delay interval compared to the treatment group (93.22 days versus 67.35 days, *p* = 0.004), however there was no correlation between delay interval and treatment interval (Spearman’s rho − 0.003, *p* = 0.979).

Table 4 shows the odds ratio (OR) results of the multilevel binary logistic regression of anti-VEGF treatment (OR 1.918125, 95% CI 1.562129–2.35525, *p* ≤ 0.001), subretinal fluid (OR 1.790373, 95% CI 1.348862–2.376403, *p* ≤ 0.001), and macular hemorrhages (OR 2.415091, 95% CI 1.916587–3.043256, *p* ≤ 0.001). The odds of receiving an anti-VEGF treatment, the presence of subretinal fluid, and developing a macular hemorrhage were greater in the post-lockdown time period compared to the pre-lockdown time period. Intraretinal fluid status did not, however, change in the post-lockdown period. This suggests that both treatment and select anatomic variables worsened after care delay.

**Table 4:** Secondary outcomes for the post-lockdown time period compared to the pre-lockdown period using a multilevel binary logistic regression model.

Parameter in post-lockdown time period	Estimate (odds ratio)	Significance	95% confidence interval
Anti-VEGF treatment	1.918125	*p* ≤ 0.001	1.562129 to 2.35525
Intraretinal fluid	1.216121	*p* = 0.183	− 1.09696 to 1.622352
Subretinal fluid	1.790373	*p* ≤ 0.001	1.348862 to 2.376403
Macular hemorrhage	2.415091	*p* ≤ 0.001	1.916587 to 3.043256

Significant estimates denote differing odds between the pre- and post-lockdown period.

## Discussion

Our data indicate that with long-term follow-up, care delay during the COVID-19 pandemic among our cohort of nvAMD patients did not significantly impact the rate of visual acuity loss when comparing historic longitudinal data to visual outcomes after care delay. Although visual acuity decreased during the study period, it was not significantly different when comparing pre- or post-lockdown time periods. However, frequency of anti-VEGF treatment and anatomic outcomes such as presence of subretinal fluid and macular hemorrhage did increase significantly after delay suggesting that although long-term visual acuity was relatively stable, a period of treatment acceleration may have been necessary to preserve vision. For instance, the odds of receiving an anti-VEGF treatment were nearly twice as great in the post-lockdown period compared to pre-lockdown. Intraretinal fluid, one of the most important markers of disease activity did not change significantly after care delay which may have contributed to preservation of vision. A brief report during the peak of the pandemic also showed an increased rate of submacular hemorrhages during April of 2020 when compared to historic data^14^.

Overall, our findings contrast with other studies that have looked at nvAMD patients with care delay due to the COVID-19 pandemic^15–18^. However, many of these studies have short follow-up or lack a proper control. For instance, Song et al. noted a decrease in visual acuity among proliferative diabetic retinopathy (PDR), DME, retinal vascular occlusion (RVO), and nvAMD patients who cancelled or no showed appointments during the mandated lockdown compared to a cohort who attended appointments, although nvAMD patients lost vision to a lesser degree than PDR, RVO, and DME patients^17^. However, use of a comparison cohort who attended clinic during the pandemic is problematic as the comparison groups (patients who attended visits versus those who missed) are inherently different. Additionally, visual acuity was compared in the pre-lockdown visit to the subsequent post-lockdown visit with average care delay being 5.34 weeks. This perhaps suggests an initial deterioration of vision, but does not examine long-term outcomes. Similarly, Zhao et al. analyzed patients with polypoidal choroidal vasculopathy (PCV) and nvAMD who had care delayed^16^. They defined a delayed cohort as lost to follow-up for at least 3 months and compared these to patients seen during the same time period with follow-up through August 2020. Although they did find a loss of visual acuity among their delayed cohort, the comparison cohort is again inherently different and likely not entirely comparable. Furthermore, the prolonged delay in care (> 3 months) and short follow-up may explain the difference in outcome between our studies. Rush et al. also used a comparison cohort of patients who were seen regularly during the pandemic versus those were delayed but looked at outcomes 6-months after care resumption and found worse visual acuity and increased central macular thickness among the care delay group^15^. Patients within our study had an average care delay of 79.5 days and follow-up of over a year which may have been sufficient time for vision to stabilize once returning to care.

Given the difficultly in determining a proper comparison cohort, we decided to use historic data from the same population as our comparison. We further longitudinally modeled our data from all visit encounters to compare rates of visual acuity change rather than a paired time comparison, as other studies have^18,19^, as it is not unreasonable to assume that visual acuity may worsen over time regardless. For instance, Elfalah et al. used paired time comparisons among 22 AMD patients who were delayed due to COVID-19 and noted a slight worsening in visual acuity on re-presentation. However, no further follow-up was reported.

Zehden et al. chose to compare historic data from a separate matched cohort of patients to patients with care delay and noted a decline in visual acuity and an increase in central macular thickness at the visit upon care resumption, but these factors stabilized by the third visit at least 4 months after care resumption^20^. Zehden et al.’s methodology helps to bypass the issues discussed above and is more consistent with our findings in that long-term outcomes appear to show a stabilization in visual acuity. Our data represent a comprehensive analysis of long-term longitudinal data among those who had care delay and accounts for patient characteristics, anatomic, and treatment factors that may help bridge the differences between previous study findings. Again, our cohort’s mean delay interval was only approximately 2 months (with a maximum of 348 days), and therefore on average those who did have care delayed were still followed relatively closely.

Interestingly, the only factor within the multivariate model that predicted a faster rate of visual decline among patients with care delay was a shorter interval of delay. This may not necessarily be a clinically significant finding as each month of care delay contributed approximately to an increased yearly loss of 1.5 letters in visual acuity (0.03 logMAR). Although this association may seem counterintuitive at first, it potentially indicates that care providers were appropriately triaging patients who cancelled, no showed, or missed appointments and correctly delayed patients who were at lower risk of visual decline when rescheduling appointments. We note in a sub analysis that patients in the active treatment group had significantly shorter care delay compared with those in the sporadic treatment/observation group. On the other hand, treatment interval was not correlated with delay interval. Although this may explain some of this association, likely, there is some other factor for which we are not able to account as delay interval was still significant even with adjustment of treatment and anatomic variables in the multilevel model. Male sex also predicted a faster rate of visual decline in our initial single-covariate model, and this trended towards similar results in the multivariate model but was not significant. This suggests that our male patients may have had worse disease status and were prone to losing vision faster.

Interestingly, with a long-term longitudinal analysis, no anatomic factor or treatment factors significantly predicted visual acuity loss. It is important to note, however, that estimates for slope effects for treatment variables did suggest that those in the treatment group or those with shorter treatment intervals lost vision at a greater rate, however, estimates were not significant. For instance, the observation group (− 0.082125 logMAR/year) would be associated with a decreased rate of vision loss of approximately 4 letters per year. Similarly, each month of treatment interval was associated with an additional loss of approximately 0.5 letter per year. Regardless, this effect size may not be clinically significant. Only 53% of our patients were in the active treatment group which represented patients with a defined treatment interval less than or equal to 16 weeks or those that re-activated/were newly exudative at the visit prior to lockdown. In line with previous research, patients who had intraretinal fluid had overall worse mean visual acuity, but this factor did not predict a greater rate of visual acuity loss.

This study had several limitations. First, it is a single-center analysis of patient data with approximately 19% of eyes being lost to follow-up post-lockdown which likely introduced bias within our model. Although we attempted to capture significant baseline and time-varying covariates that could influence visual acuity, anatomic variables including subretinal fluid and intraretinal fluid were graded in a binary fashion based on the provider’s note. Likely, central macular thickness would have been a useful marker to study. Lastly, it is important to note that failure to achieve significance does not necessarily mean the association is not present the population, it simply means data from our sample could not reject the null hypothesis of no significance.

## Conclusions

This is the first study to longitudinally model nvAMD patient visual acuity and anatomic outcomes longitudinally with long-term follow-up among patients who had their care delayed due to the COVID-19 pandemic. It suggests that in contrast to shorter term studies, visual acuity was not significantly impacted by the mandated lockdown period although both anti-VEGF treatment increased, and some anatomic factors worsened after care delay. No single demographic factor was predictive of visual acuity loss in patients with care delay. Although the COVID-19 pandemic has officially ended, our study suggests that short-term care delays in the context of other emergent events or simply issues with patient follow-up may be acceptable but may come at the cost of increased need for treatment post-delay. These conclusions need to be taken in the context of our cohort population and the above discussed limitations.

### Supplementary Information

Supplementary Information 1.

Supplementary Information 2.

Supplementary Information 3.

## Data Availability

All data generated or analyzed during this study are included in this published article (and its Supplementary Information files).
