# Supplementary material for: Clinical outcomes in neovascular age-related macular degeneration: a cohort study of patients with care delay due to the COVID-19 pandemic
Source: Sci Rep. 2023 Sep 8;13:14814. doi: 10.1038/s41598-023-41497-4 (PMC10491764; doi:10.1038/s41598-023-41497-4)
Supplement: Supplementary file 3 — Supplementary Information 3. [file 41598_2023_41497_MOESM3_ESM.docx]

Dependent: Visual acuity (VA) – continuous, logMAR
Covariates:

| Variable | Level | Description | Values |
| --- | --- | --- | --- |
| EyeID | Level 1 | Eye identifier across 2 years | Integer |
| PatientID | Level 2 | Individual identifier across 2 years | Integer |
| Eye |  | Right = 1, Left = 2 | 1, 2 |
| DateofEncounter |  | Date of individual encounter | Date |
| Time | Level 1 | Integer corresponding to linear date 3/1/2019 (0) to 7/1/2021 | 0, 1, 2, 3… |
| Timepre | Level 1 | Integer corresponding to linear date 3/1/2019 to 3/13/2020 | 0, 1, 2, 3…422, 422, 422, 422 |
| Timepost | Level 1 | Integer corresponding to linear date 3/14/2020 to 7/1/2021 | 0, 0, 0…0, 1, 2, 3… |
| Timequad | Level 1 | Time^2 | 0, 1, 4, 9… |
| AntiVEGF (treatment for AMD) | Level 1 | Received injection at visit (1) | 0, 1 |
| Subretinalfluid (marker of disease activity) | Level 1 | Indicates presence of subretinal fluid at visit | 0, 1 |
| Intraretinalfluid (marker of disease activity) | Level 1 | Indicates presence of intraretinal fluid at visit | 0, 1 |
| Geographicatrophy (marker of disease activity) | Level 1 | Indicates presence of geographic atrophy at visit | 0, 1 |
| Macularhemorrhage (marker of disease activity) | Level 1 | Indicates presence of macular hemorrhage at visit | 0, 1 |
| Timeperiod | Level 3 | 0 = pre-covid  1 = post-covid | 0, 1; dummy variable |
| Age | Level 3 | Mean age of patient | Scale |
| Sex | Level 3 | Sex | 0=male, 1=female |
| Race | Level 3 | Race | 0=Other declined  1=White  2=AA  3=Asian  4=Pacific Islander |
| Treatment (some patients require active treatment, others if stable or treatment is futile are observed) | Level 2 | 0 = Observation  1 = Active treatment / converted during study period | 0, 1 |
| Treatmentint (treatment requires monthly to up to 16 week eye injection; each patient has a unique treatment interval) | Level 2 | Treatment interval pre-covid in days | Integer |
| Delayint | Level 2 | Delay interval in care in days (date o follow-up - appointment date canceled) | Integer |
| LTFU (Lost to follow up) |  | 0 = Follow-up scheduled  1 = No follow-up | 0, 1 |
| Deceased |  | 0 = Alive  1 = Deceased during follow-up | 0, 1 |
| AntiVEGFtype | Level 2 | Type of agent used for treatment (visit prior) | Factor 0 = none (observation)  1 = Eylea  2 = Lucentis  3 = Avastin  4 = Multiple / other or study drug |
|  |  |  |  |
| Subretinalfluid_baseline | Level 2 | Baseline subretinal fluid pre-covid | 0, 1 |
| Intraretinalfluid_baseline | Level 2 | Baseline intraretinal fluid pre-covid | 0, 1 |
| Geographicatrophy_baseline | Level 2 | Baseline geographic atrophy pre-covid | 0, 1 |
| PriorPDT (a one time treatment that can be effective in decreasing disease severity) | Level 2 | Previous PDT treatment | 0, 1 |
| Smoking (only known modifiable risk factor for AMD development) | Level 3 | 0 = Never smoker  1 = Former  2 = Current | Factor |
